# Dr. Andrews, on Vaccination at Madeira

**Published:** 1810-02

**Authors:** 


### To Dr. ADAMS.

My dear Sir,

I Had hoped that sufficient proof was already adduced, not only of the benefits to be derived from that source, but of the great advantages attending the introduction of vaccination over the inoculated small-pox, as at least to have satisfied medical men, and to have fully justified the interference of the legislature, in preventing a continuance of the latter practice, as far as such an interference could be effectual.

There still appear to be *some*, who think proper to doubt the probity of the College; whose interest, in determining the point in question, could only be directed to the public good.

Of such a class is Dr. Carneiro; and of how little soever importance his work may be in the opinion of professional men, yet with the public at large, it is calculated to shake the confidence of those, whose only power of determining, is by comparing what may be advanced by the partisans on either side. Under such circumstances, therefore, it becomes the duty of every individual to correct whatever he finds erroneously stated; and to that part of the book which bears reference to this island, I shall confine my reply.

It is evident that the Doctor speaks only from report, but this is not a sufficient apology, as he should have taken pains to correct or confirm his information.

He says, "Besides, we learn from travellers and correspondence, that in the Island of Madeira, where the vaccine influence has been so prevalent, an eruptive fever, called by the physicians and surgeons measles, has raged epidemically and fatally.—Here it may be observed,

"1st. That the persons attacked with the eruptive fever, had the measles before.

"2dly. That the eruptive fever now predominant, has not so regular a type as the true measles.

"Hence it appears, that the diseases most frequently consequent on vaccination in warm climates, are of a pituitous kind, called by the cow-pox partisans, measles, having no other symptoms of the true measles, than the sneezing and the smallness of the eruption."*

That the epidemic alluded to, and which the Doctor is pleased to consider as an eruptive fever, the consequence of vaccination, was measles, will, I trust, be satisfactorily proved; and that vaccination has been successfully practiced here, I am equally sanguine that the evidence I shall produce will sufficiently establish.

In the beginning of May, 1808, a detachment of the 11th regiment landed here; some of the soldiers' children, brought on shore with the measles on them, were taken to the Military Hospital.

A few days after their landing, I was desired to see a child of Mr. Cathcart's, (the American Consul for this Island,) who had a considerable fever; in two days an eruption appeared, accompanied with all the symptoms of measles distinctly marked. At this time, however, though confident

* Vide Reflections and Observations on the Practice of Vaccine Inoculation, &c. by H. Carneiro, M. D. p. 88 and seq. English Edit.

confident that the disease was measles, I was unacquainted that it had been introduced by the troops; and on my mentioning the case to Dr. Gourlay and others, they were rather led to think I might have been mistaken in the disease. It, however, went through the whole of the family, consisting of five children, one of whom was so severely affected with the pulmonary symptoms, as to require blood-letting to a considerable extent. A lapse of near two weeks took place, before another *island*-case appeared, and this also happened to be a patient of mine, living in the neighbourhood of the former. This led me to trace the source of infection; and it was then that I learnt the circumstances above related, and found that the little girl, who was the subject of the last case, had been in the habit of looking into the court-yard of the hospital as she passed by to go to school; and as the servants of the Consul daily passed the hospital gate in their way to and from town, I felt no difficulty in concluding that the infection might have been conveyed by those means.

It was at first objected, that the great length of time since that disease had visited this island before,* rendered it likely that it would have immediately spread, with a rapidity that such diseases are noticed to do in warm climates, and consequently, that the other practitioners would, ere this, have seen some cases also. This, however, happened so soon afterwards, as to put the matter beyond a doubt.

That the measles terminated fatally in many cases, there can be no doubt; it is not less certain, that neither the disease nor its consequences could be attributed to the effects of vaccination, since many who had it, never were vaccinated, but had regularly gone through the inoculated or casual small-pox.

Although the great mortality of that year was generally attributed to the measles, I cannot help considering them in a great proportion of cases, as the predisposing cause only; the fatal cases having generally terminated by dysentery, and that at a considerable distance of time, after the measles had been considered as subdued in those particular cases to which I allude.

To persons who have resided here, it is well known, that during the *fruit* season, there is generally a bowel complaint prevalent, arising from the too free use of

 that

* I believe about fifteen years.

that *article* in all its stages of maturity. The natives of the lower class, in fact, eat every part of the fruit, never considering whether it be ripe or not; the consequence of such a *diet* (for in reality it is *such* to many), after the constitution had been previously reduced by a severe inflammatory disease, was, what a priori might have been expected, acute dysentery, which in a great many instances terminated fatally.

As an additional proof of what I have advanced, it may be observed, that when a proper attention had been paid to the state of the bowels, for some weeks after the measles were subdued, and no fruit or irritating matter of any kind had been received into the stomach, such patients universally recovered.*

With respect to the effects of vaccination, as rendering the constitution free from the susceptibility of small-pox contagion, we have something more than presumptive evidence.

In June 1808, when the 2d Royal Veteran battalion arrived here, it was reported that they had some children under the influence of small-pox; of course, there was much apprehension entertained of its spreading.

I did not then consider that vaccination had been practiced with such energy by the professional men here, as appears really to have been the case.

About eight or ten days after the troops had landed, whilst in conversation with Dr. de Fretis and Mr. Woolriche on this subject, the wife of one of the soldiers came up to us, having in her arms a child with a very full crop of variola of the distinct kind, and in the latter stage. Some days after, the disease appeared upon another child, so that there can be no doubt of its having been in the island, and of its having gone through all its stages here.

As no steps were taken to prevent the infection spreading,† and as it was at the most favourable time of the year for such a circumstance, its having ended with these cases, becomes as strong an evidence in favour of vaccination, and as great a proof of the activity of the medical men of the island, as can be required.

Still, without a bond of union, or an establishment for the

---

* It is to be remembered that I speak more particularly of such cases as came under my own immediate observation, but I have also heard the same remark from others.

† The children were allowed to mix with the natives.

the preservation of the matter, it is not wonderful that it should become exhausted, the exertions of individuals are unequal to the charge; besides, that, from a particular religious impression, many of the natives are averse to vaccination.* This induced me, in the year 1805, to propose to the governor, Senhor Arcaneo da Seguiera Freire, to form an establishment under his patronage for the general inoculation of the cow-pox through the island; and in order to render it more effectual, to obtain the assistance of the clergy, in removing their scruples concerning interfering with the *Divine Will.* I was particularly led to make this proposal, from having been informed that the governor had received instructions from his court, to give every assistance in his power to the cause.

Well aware that a plan upon an expensive scale was not likely to be countenanced, I had arranged it so that the government would not have been put to any expence; some of the merchants to whom I communicated my intentions, readily offering to become subscribers.

My proposals, however, met with no encouragement, and consequently every thing was left to depend on the zeal of individuals.

Shortly after the present governor arrived, I repeated my applications to him, but with the same ill success. Had General Beresford continued longer in the government, he promised me every assistance in his power.

Not having kept a register, I cannot with any accuracy state the number that I have vaccinated, but suppose, since 1803, it cannot amount to more than from four to five hundred, and I cannot recollect a single instance of any ill effect. Many of the number may have failed, as I had it not in my power to ascertain the progress of the complaint in those who lived at a distance from the town, as those children were most frequently not brought to me again, after the operation had been performed. In one or two cases, an ulcer on the arm has been the consequence of an early removal of scab, but this readily yielded to a weak solution of vinegar and water.

As however it is desirable in the cause of truth, wherein the comfort and happiness of so many depend, that no part of the report should rest on the authority of any individual, I wrote the following circular note to Dr. Gourlay,

* This is principally confined to the lower class.

lay, Dr. De Fretis, physico mor, in the island of Madeira, &c. and Dr. Curado; their answers to which I have subjoined.

*(CIRCULAR.)*

*To Dr.* WILLIAM GOURLAY, *Physician to the Factory in the Island of Madeira.*

DEAR SIR,

YOU will oblige me by the favour of your answers to the following questions, and such further observations as you may have made on vaccination.

Believe me, yours truly,

*Funchall, Oct* 6, 1809. M. W. ANDREWS.

1st. In the course of your practice, did you ever meet with any unpleasant symptoms that you could attribute to vaccination? If you have, what were they?

2d. Are you aware of any unpleasant occurrence that has taken place in any of the patients you vaccinated, immediately after, or at any subsequent period? If so, what is it? and can you attribute it to that cause?

3d. Do you believe the epidemic that was so prevalent here in 1808, to have been the measles? or do you consider it to have been an eruptive complaint induced by vaccination?

4th. Do you recollect if any persons you vaccinated had the measles afterwards? If so, did they appear to suffer more, or were they in greater danger on that account? or did the proportion of bad cases of measles, appear to depend upon that cause?

5th. About what number of persons have you vaccinated since 1803?

Here follows Dr. Gourlay's answer; Dr. Antonio C. de Fretis's answer, and Dr. Angelo Curado de Meneges, with translations.

To Dr. M. ANDREWS.

SIR,

In compliance to your request, by favouring you with answers to questions, and such further observations which I have made on vaccination, I hope the following will meet your approbation.

I am, &c.

*Funchall, Oct* 6, 1809. W. GOURLAY.

In reply to your first and second questions, I do not recollect of meeting with any unpleasant symptom of consequence, quence, which I could attribute to vaccination; nor am I aware of any unpleasant occurrence, either immediately after or at any subsequent period, which I could ascibe to that cause.

In regard to your third question, I am of opinion, that the epidemic which was prevalent here last year, was measles; the eruption not induced by vaccination; and which had been imported from England by a detachment of the 11th regiment; the nature of which I refer to a work which will soon be published, "Observations on the endemial and epidemial diseases in Maderia, &c."

In answer to your fourth question: Numbers of those persons whom I had vaccinated, laboured afterwards under measles; but I am not sensible that they suffered more, or were in greater danger on that account: nor did the proportion of bad cases appear to depend at all on that cause.

Your fifth and last question I have to reply; that to the best of my remembrance, the number of persons I have vaccinated, have not exceeded four hundred.

### From Dr. ANTONIO C. De FRETIS.

(Translation.*)

THE practice of Vaccination, which, since the year 1805, has been exercised in this island to the greatest extent under my direction, and assisted by the government, agreeable to the orders of H. R. H. to make it general in this colony, enables me to answer the questions which you have proposed, with propriety and correctness.

In the year 1805, not less than 2000 persons were vaccinated by me, and those people whom I delegated for that purpose in the different parts of the island, without the loss of one; nor have any subsequent symptoms appeared after that operation, which have made me repent the practice.

In the greater part of those vaccinated, the eruptive fever was scarcely perceptible; some few experienced a slight degree of weakness for a day or two, others continued in their ordinary occupations during the whole course of the complaint, almost insensible to its effects on the constitution, nor did the eruption extend itself beyond the inoculated part, except in one child, seven years old, who had a tolerable smart fever, and had 25 eruptions in different

* The original is transmitted to the Editors.

ferent parts of its body, each about the size of a sixpence. This child, however, recovered without any further inconvenience.

I inoculated a child, daughter to Sen. Francisco Anacleto Figueirao, lieut. col. in the militia, three years old; she passed through the complaint tolerably well, but on the falling off of the scabs, deep ulcers remained in the parts where the incisions were made, requiring a greater length of time than usual to cicatrize.

A person, of little reflection, would possibly attribute this to the virulence of the vaccine virus, it is therefore necessary to say, that I observed the same circumstance in her sister, who, two years before, had been inoculated with small-pox, and in whose arms very large cicatrices are still remaining.

From what I have related, you will see that the eruptive process has always been mild; that vaccination has not failed in any; nor do I recollect that this operation has been followed by any disease that could be attributed to that as a cause.

Two epidemics prevailed in this colony in the year 1808, introduced by the British troops forming the expedition commanded by General Beresford.

The first, and which spread immediately after the troops landed, was the hooping-cough; this epidemic destroyer of so many individuals in this colony on that occasion, equally attacked those persons who had been vaccinated, and those who had not undergone that operation. Many of those who had been vaccinated recovered, and others who had not, died, as if proving the inverse of what had been asserted, that the fatality was equal with both classes.

The second epidemic was the measles, introduced about three or four months after the former had been got under, by a detachment which came to complete one of the regiments of the afore-mentioned expedition. Some of the children with this detachment when they landed, were said to have the measles, which was confirmed, not only by the army medical gentlemen, but by the resident practitioners. In a short time this contagion spread through the city and its environs; at first the disease appeared in a mild form, but in its progress it became terrible, and its consequences produced considerable mortality throughout the island.

The first family that I attended with this disease, was that of Sen. Antonio José Spinola, Captain in the militia, whose whose wife and eight children suffered from it. This lady was the first victim to the complaint.

The eruptive stage passed off without any extraordinary symptoms, and she left her bed, considering herself as perfectly recovered. She attended her children in the complaint for some days, and when it was least expected, had a pneumonic attack, which suddenly terminated in death. She had not been vaccinated; all her children had, and with the exception of *one*, recovered.

About the same time, a Lieutenant of the regular troops, Sen. Joas Bittancourt, a strong, robust person, who had never been vaccinated, received the infection, and went through the disease with the ordinary symptoms, till the eighth day; when considering himself recovered, he left his room. A diarrhœa, however, supervened, and in twenty-four hours he died.

Senhora Donna Maria Antonia Correa, a lady well known in this town for her virtues and benevolence, not previously vaccinated, was attacked with measles; she had the ordinary symptoms until the seventh day, when a diarrhœa came on with such violence, that she was for a considerable time in great danger, and the consequent debility remained so long after the disease was removed, that it was with difficulty her constitution recovered from its effects.

A grand-daughter of this lady, who had undergone vaccination, recovered from the measles, without any unpleasant symptom, and her sister, who had not been vaccinated, also went through the measles without any unusual occurrence. Three sons of Captain John de Fretis, who were vaccinated a short time before, had the measles; two died in consequence of the diarrhœa which supervened, and the other recovered.

I could adduce many other examples, but those I have noticed, are people well known in the island.

By the regiment that landed here to relieve the 11th, who are now serving in Portugal, the natural small-pox was introduced; several children in the eruptive stage of the complaint, passed through the streets without having communicated the disease to any of the inhabitants; this must be attributed to the effects of vaccination, as on the former occasion, when the small-pox was introduced, it spread rapidly through the whole island.

From what has been said, we may infer, 1st. That vaccination appears to have rendered the inhabitants of this island free free from the contagion of the natural small-pox; which was recently introduced by the present garrison.

2dly. That it has not produced any bad consequences either immediately after, or at any subsequent period, (up to the present date,) to those persons who have regularly gone through the process; and,

3dly. That the epidemics did not attack those who had been vaccinated with more violence, than those who had not undergone that operation.

I am, &c.

ANTONIO C. De FRETIS.

To Dr. ANDREWS.

(Translation.) The original with the Editors.

Dear Sir,

In consequence of the irregular manner in which vaccination has been practiced in this island, it will not be in my power to give you such satisfactory answers to the questions you have proposed as I could wish; the following, however, are such as occur.

1st. I have never met with any unpleasant symptom in my practice, that could be attributed to vaccination.

2d. I do not remember having seen any of those bad consequences that have been imputed to vaccination; I have only met with three or four children, in whom an itchy cutaneous eruption, somewhat resembling urticaria, appeared about ten or sixteen days after vaccination, but which easily yielded to simple diaphoretic medicine. These exanthemata have been commonly considered as the effects of cow-pox, but I by no means think so. One of the children was teething, and the others had been subject to cutaneous eruptions before*, but in a mild degree; and the parents are not the most healthy. Besides which, cutaneous diseases are very common in many families of this island.

3dly. The epidemic which prevailed here last year, and which was brought by an English ship, had the Nosological characters of the true measles, resembling those that prevailed about the same time in England and Scotland, as described by Dr. Ferguson in the Medical and Physical Journal,

* Two of those alluded to by Dr. C. the one that was teething, and one of the others, I vaccinated, at which time the eruption was out; one of them was vaccinated twice, before it took effect.

Journal, No. 123. Independent of which, the first people whom I saw in the complaint, had not been vaccinated; many people of 70 years of age, who had before escaped the infection, and on that account supposed they had gone through the disease, on this occasion suffered much.

4thly. Many children, who had been vaccinated some years before, and others a short time previous to the epidemics appearing, also had the measles; some of these dangerously, but by far the greater number had the complaint in a very mild way. The worst kind of measles, and the greatest mortality, were to be met with amongst the poorest people.

I attribute this to their situation, not having the means of supplying themselves with medicines, and a proper diet; and also to the indiscreet religious maxim, which teaches them to consider the death of their children in their infant state as a great blessing.

5thly. I cannot with exactness say the number that I have vaccinated, having sent the greater part of those who applied to me, to be vaccinated by Sen. Antonio da Silva Silveira; who, from 1804 to the present date, has vaccinated more than four thousand.

I am, &c.

Dr. Joas Angelo Curado de Meneges.

I also wrote to Mr. Woolriche, surgeon to the forces, and principal medical officer in the island, requesting such information on these subjects as his situation enabled him to afford me. To which I received the following answer.

To Dr. ANDREWS.

Dear Sir,

In answer to your inquiries relative to the progress of vaccination in this garrison, and the nature of the prevailing epidemics of last year, I shall shortly state what I happen to know on the subject.

Soon after the arrival of the British troops here in 1807, the vaccine virus was procured from England; from neglect, or bad arrangement, it soon was lost, and several months elapsed before a supply could be procured; subsequently, however, it was employed with so much industry and success as to include, I believe, nearly all that were liable to receive the variolous infection*. The Portuguese physicians

* In the families of the Military.

physicians too, appear to have been active, as a circumstance I shall mention immediately, strongly proves.

That the prevailing epidemic of last year was measles, I have not the slightest doubt; the cases I saw were clearly marked; in many instances, the pulmonary affections were very severe at the beginning of the disease, and obstinate bowel complaints, with bloody stools, were common at the termination of it. If there could be a question on this subject, the way in which the disease was introduced, I think would decide it. Early in May 1808, a detachment of the 11th infantry arrived from England; amongst the sick some cases of measles were reported; circumstances, however, rendered it necessary to empty the transport immediately, and the disease was established in the island. What I alluded to above, as proving the extent to which vaccination has been carried here, was the arrival of the 2nd Royal Veteran Battalion in June last, having amongst their children several cases of small-pox, which were landed without my knowledge; my anxiety was excessive, on meeting some days afterwards in the street, one of the children covered with pustules; it seemed too late to do any thing effectual, and in fact, nothing was done to prevent the spreading of the disease.

A younger sister of one of these children was afterwards attacked, she had an extensive eruption of distinct pustules, and here the disease ended, as far as I know, not having heard up to this date of any other case; a tolerable satisfactory proof, I think, of the activity of the medical people here, and of the efficacy of vaccination.

I am, &c.

S. WOOLRICHE, P. M. O.

*Funchall, Oct.* 19, 1809.

